# Analgesic drug use in patients with STEMI: Current perspectives and challenges

**DOI:** 10.3389/fmed.2023.1148581

**Published:** 2023-03-22

**Authors:** Huaigang Chen, Hong Wang, Bin Li, Lang Hong, Maobin Kuang, Liu Yang

**Affiliations:** ^1^Medical College of Nanchang University, Nanchang, Jiangxi Province, China; ^2^Department of Cardiology, Jiangxi Provincial People's Hospital, The First Affiliated Hospital of Nanchang Medical College, Nanchang, Jiangxi Province, China

**Keywords:** STEMI, P2y12 receptor antagonists, morphine, analgesic, lidocaine

## Abstract

Therapy for patients with ST-elevation myocardial infarction (STEMI) has been a controversial topic since the introduction of thrombolytic agents in the 1980s. The use of morphine, fentanyl and lidocaine has increased substantially during this period. However, there is still limited evidence on their advantages and limitations. In this review, the clinical application, as well as future considerations of morphine, fentanyl and lidocaine in patients with ST segment elevation myocardial infarction were discussed.

## 1. Introduction

Ischemic heart disease, such as coronary heart disease, is currently the most common cause of death globally, and its incidence is rising (1). Among them, ST-segment elevation myocardial infarction (STEMI) is one of the most dangerous subtypes of coronary heart disease. In the latest STEMI practice guidelines, a loading dose of dual antiplatelet therapy (DATP), an early start of reperfusion therapy, relief of ischemic chest pain and anxiety are emphasized (2). It is important to use Anti-platelet drugs and analgesics before hospitalization, in combination with percutaneous coronary intervention, to relieve chest pain. Commonly used analgesic drugs include morphine, fentanyl, and lidocaine, but using these drugs may reduce the anti-platelet aggregation effect of oral P2Y12 receptor antagonists (Table 1) (3), which may increase the risk of outcome adverse events.

**Table 1:** Basic information for the studies of interactions between analgesics and P2Y_12_ receptor antagonists.

Study	Year	Design	Patients	Analgesic drug	Dose	P2Y_12_ Antagonist	Conclusion
RAPID	2013	Randomization	50	Morphine	NR	Ticagrelor	Morphine is an independent predictor of the 2-h HRPR
		Prospective				Prasugrel	
ATLANTIC	2014	Randomization	1862	Morphine	NR	Ticagrelor	Morphine appeared to delay the antiplatelet effect of Ticagrelor
		Multicenter					
		Double blind					
RAPID2	2014	Randomization	50	Morphine	NR	Ticagrelor	Morphine is an independent predictor of HRPP (at 1 h after loading dose) and baseline level PRU values
		Prospective				Prasugrel	
MOJITO	2014	Randomization	82	Morphine	NR	Ticagrelor	Crushing Ticagrelor inhibited platelets earlier than the whole tablet, but could not completely eliminate the inhibitory effect of morphine.
		Prospective					
		Multicenter					
Hobl et al.	2014	RandomizationDouble blind	24	Morphine	5 mg/iv	Clopidogrel	Morphine affects the absorption of clopidogrel, delaying it and reducing the area under the curve of its active metabolite by 34%.
IMPRESSION	2015	Randomization	70	Morphine	5 mg/iv	Ticagrelor	The total exposure of Ticagrelor and its active metabolites was reduced by 36 and 37%, respectively, due to the administration of morphine.
		Single center					
		Double blind					
Parodi et al.	2015	Retrospective	300	Morphine	4 mg/iv^a^	Ticagrelor	No difference in the inhibitory effect of morphine on prasugrel and Ticagrelor
						Prasugrel	
Zeymer et al.	2015	Observational	75	Morphine	NR	Clopidogrel	The platelet response index of clopidogrel after a 2-h loading dose was higher in the morphine group
						Prasugrel	
Farag et al.	2016	Observational	100	Morphine	NR	Clopidogrel	The use of morphine is associated with significantly increased platelet reactivity before interventional therapy
PACIFY	2017	Randomization	70	Fentanyl	NR	Ticagrelor	Fentanyl resulted in a delay in the absorption of Ticagrelor
		Single center					
PERSEUS	2022	Randomization	56	Fentanyl	50–100 mcg/iv; 4-8 mg/iv	Ticagrelor	No difference in inhibition of Ticagrelor by fentanyl and morphine after 2 h of Ticagrelor loading dose
		Prospective		Morphine			
		Single center					
LOCAL	2021	Randomization	70	Fentanyl	b	Ticagrelor	Plasma levels of Ticagrelor (loading dose) were significantly lower in the fentanyl group than in the lidocaine group after 2 h.
		Prospective		Lidocaine			
		Single center					
		Double blind					
AVOID-2	2021	Randomization	300	Lidocaine	c	NR	Comparison of analgesic effects and safety of fentanyl and lidocaine
				Fentanyl			
ON-TIME 3	2022	Randomization	195	Fentanyl Acetaminop-hen	NR	Ticagrelor	Iv acetaminophen in comparison with iv fentanyl was not associated with significantly lower platelet reactivity in STEMI patients, but resulted in significantly higher ticagrelor plasma levels and was effective in pain relief.
		Prospective					
		Multicenter					

HRPR, high residual platelet reactivity; PRU, platelet reactive unit; NR, not reported; iv, intravenously. a, Median total morphine dose per patient was 4 (2–6) mg with a range of 2 to 12 mg. b, Give fentanyl 0.75 mg/kg intravenously at the start if under 70 years of age, otherwise give 0.5 mg/kg. Lidocaine 1 mg/kg intravenously at the start (maximum dose 100 mg). c, To start, give 50 mg of lidocaine intravenously over 2 min if the patient weighs < 70 kg, otherwise give 100 mg over 2 min. 50ug of fentanyl given intravenously every 5 min.

The use of relevant analgesic drugs in patients with STEMI can have a significant impact on prognosis and should be carefully evaluated. This article reviews the studies on the combination of P2Y12 receptor antagonists and analgesics in patients with STEMI. In the meantime, the clinical application and important points for attention of analgesics were discussed.

## 2. Methods

### 2.1. Literature search

Retrieval should be conducted using PubMed, EMBASE, and Sino Med databases. Search terms pre-defined in titles, abstracts, and keywords are used to identify pertinent studies. More information about the terms used in the search can be located in Supplementary material 1. The retrieval period spans from the inception of the databases up to February 2023.

### 2.2. Data extraction

After independently reviewing the literature, the two researchers gathered data manually, including the type, year, drug subjects, and results of the study. To ensure impartiality, a third researcher was selected to evaluate any differences of opinion.

## 3. Application of analgesics in patients with STEMI

### 3.1. Morphine

As a potent opioid drug, morphine produces significant analgesic and sedative effects by stimulating the action of the endogenous anti-pain substance enkephalin and activating opioid receptors in the central nervous system (4). In 1930, Moor et al. reported for the first time that intravenous morphine was used to relieve pain in patients with acute myocardial infarction, and the analgesic effect reached its peak in a few minutes (5). Yet, as research on the effects of morphine continues, more and more adverse effects are being discovered, such as gastrointestinal reactions, bradycardia, hypotension, and respiratory depression (6).

Intravenous morphine may reduce the efficacy of antiplatelet medications in patients with acute myocardial infarction, especially those with STEMI. In 2013, Parodi and colleagues compared the effect of prasugrel and ticagrelor in patients with STEMI undergoing percutaneous coronary intervention (RAPID study) (7). The findings indicated that only 50% of patients achieved effective platelet inhibition 2 h after the loading dose, whereas it took a minimum of 4 h for most patients to reach effective platelet inhibition. Intravenous morphine may cause delayed antiplatelet effects of Ticagrelor or prasugrel. Researchers believe that morphine use is a predictor of high residual platelet reactivity (HRPR). The results of the ATLANTIC study concluded that, in patients with STEMI, prehospital administration of Ticagrelor in the short term before PCI appears to be safe but does not improve coronary reperfusion before PCI. One potential reason for the delayed absorption of ticagrelor may be due to the drug interaction with morphine. However, this has not been further clarified in this experiment (8).

The results of the RAPID-2 study demonstrated that a greater load dose of ticagrelor could result in more efficient and quicker platelet inhibition. It was also proved that morphine was an independent predictor of HRPR at 1 h after loading dose (OR: 4.49 [1.19–16.88], *p* = 0.026) and platelet reactive unit (PRU) values (OR: 1.015 [1–1.03], *p* = 0.039) (9). The inhibitory effect of morphine on antiplatelet agents is most pronounced at the time of initial administration. To further determine the difference in the negative effects of morphine among antiplatelet drugs, Parodi et al., conducted a study in 300 patients with PCI who received loading doses of prasugrel or ticagrelor. Platelet reactivity was evaluated by Verify Now at 1, 2 and 4 h after loading. Patients who were treated with morphine were found to have higher rates of vomiting. There was no difference in the inhibitory effect of morphine on prasugrel and ticagrelor (10). In addition, the MOJITO study found that crushing ticagrelor inhibits platelets earlier than taking the drug whole. However, even with this faster reaction time, using morphine can increase the reactivity of residual platelets (11). The TASTER study, led by Guido Parodi et al., examined the effectiveness of a new Ticagrelor formulation, an oral dispersible tablet (ODT) that does not require water and quickly releases its components upon swallowing. Despite the lack of significant distinction between the new orally disintegrating tablet (ODT) and the conventional Ticagrelor tablets in terms of platelet inhibition, ODT has a superior safety and convenience advantage, particularly in ambulances (12).

However, most of the previous studies were retrospective, observational, or did not focus on morphine. The IMPRESSION trial is the first randomized trial to confirm the negative effects of morphine on the pharmacokinetics and antiplatelet effect of ticagrelor in patients with AMI (13). Patients were given an intravenous dose of either morphine (5 mg) or placebo, followed by a load dose of ticagrelor (180 mg). The results showed that morphine reduced the total exposure of ticagrelor and its active metabolites by approximately one-third (AUC (0–12): 6307 vs. 9,791 ng h/ml; *p* = 0.003) and (AUC (0–12): 1503 vs. 2,388 ng h/ml; *p* = 0.008).

Clopidogrel is another P2Y12 receptor antagonist that is frequently used in the clinic. According to Hobl et al., morphine has the potential to reduce the concentration of clopidogrel and its active metabolite, as well as delay their absorption (14). Morphine can delay the inhibitory effect of platelet occlusion at high shear rates, thereby prolonging the occlusion time caused by collagen diphosphate by 3 times with its active metabolite. Zeymer and Farag et al. also demonstrated that morphine can delay the platelet inhibition of clopidogrel (600 mg) (15, 16). In addition, Farag also found that the delayed effect of morphine on clopidogrel disappeared after 24 h, which may be related to the metabolism of morphine from the body. A meta-analysis of 207 STEMI patients from 5 studies found that morphine caused an approximately 40% increase in expected platelet reactivity (*p* < 0.001) (17).

### 3.2. Fentanyl

Fentanyl is a potent, rapidly administered synthetic opioid that is injected intravenously (18). In recent years, an increasing number of studies have reported the effect of fentanyl in early analgesia in patients with acute myocardial infarction. The effect of fentanyl on the blood concentration of a P2Y12 receptor antagonist has not been well studied. It is possible that fentanyl could reduce the concentration and delay the antiplatelet effect. John and his colleagues first reported in the PACIFY trial the effect of intravenous fentanyl on ticagrelor absorption and platelet inhibition in patients receiving PCI (19, 20). The results showed that the incidence of high residual platelet reactivity and platelet aggregation was significantly higher in the intravenous fentanyl group than in the non-fentanyl group (20% vs. 6% and 33% vs. 5%).

The effects of fentanyl and morphine on the absorption of a loading dose of ticagrelor (180 mg) and on platelet inhibition were compared in patients with STEMI by Sophie et al. (21, 22). Fifty-six patients were randomly assigned to either the group receiving morphine or the group receiving fentanyl at a 1:1 ratio. Subsequently, the platelet reactivity to Ticagrelor was recorded 2 h after the loading dose and evaluated by P2Y_12_ response units (PRU). The results of the study showed that fentanyl can have an adverse effect on platelet inhibition after 2 h of ticagrelor loading dose comparable to morphine (173.3 ± 89.7 vs. 173.3 ± 89.7, *p* = 0.179). After 4 h, the patients who were treated with fentanyl had significantly lower PRU values than the patients who were treated with morphine (90.1 ± 97.4 vs. 168.0 ± 72.2; *p* = 0.011). This may indicate that the inhibition of ticagrelor by fentanyl is short-lived, compared to morphine. In the end, the results of this study are inconclusive, leaving it unclear whether fentanyl is a better option than morphine. Furthermore, the lack of assessment of pain outcomes between the fentanyl and morphine groups in this study is a limitation. Consequently, the actual clinical implications of this variation require additional investigation.

### 3.3. Lidocaine

The exploration of non-opioid analgesics has provoked comprehensive interest among scholars. Lidocaine is a sodium channel blocker that is usually used for local analgesia and local anesthesia, as well as for the treatment of ventricular arrhythmias. Prior investigations have demonstrated that lidocaine is efficacious in reducing ischemic pain, including among patients with coronary artery disease (23, 24). Lidocaine produces analgesia by interfering with the function of sodium channels and G proteins, which in turn prevents activation of N-methyl-D-aspartic acid receptor (NMDA). It can reduce circulating inflammatory cytokines, preventing secondary hyperalgesia and central hyperalgesia (25).

In the LOCAL trial, the study by Himawan et al., demonstrated the safety and efficacy of lidocaine for analgesia in patients with coronary artery disease. Moreover, it will not impair the bioavailability of ticagrelor or delay its antiplatelet effect, compared to fentanyl (26). In this study, it was found that intravenous lidocaine and fentanyl have comparable analgesic effects (pain assessment by 11-point numerical rating scale -NRS). Both treatments receive high marks from patients in terms of satisfaction. The study under discussion is a PK/PD study of a certain drug, rather than a clinical study of outcomes. Thus, it is difficult to ascertain the clinical significance of the findings of this study. In addition to this, patients with unstable angina pectoris or non-ST segment elevation myocardial infarction were the only patients included in this study. In the AOVID-2 trial, 300 patients who were suspected of having STEMI were randomly assigned to receive either intravenous fentanyl or lidocaine in the emergency ambulance in order to relieve chest pain (27). Patient analgesic effect (pain reduction on arrival at the hospital) and safety (e.g., adverse drug reactions) were the co-primary endpoints of this study. The findings of the recent study have revealed that lidocaine does not meet the criteria for non-inferior efficacy, and the effect of prehospital analgesia is inferior to that of fentanyl. However, lidocaine is safer and better tolerated than intravenous fentanyl (28). Interestingly, the study’s secondary endpoint also compared the relationship between the size of the infarct and the dose of analgesics in the two groups. A dose-dependent relationship between opioids and the size of myocardial infarction has been reported in previous studies (29). The administration of analgesic drugs may need to be more individualized in patients with myocardial infarction.

## 4. Discussion

**Figure 1:**
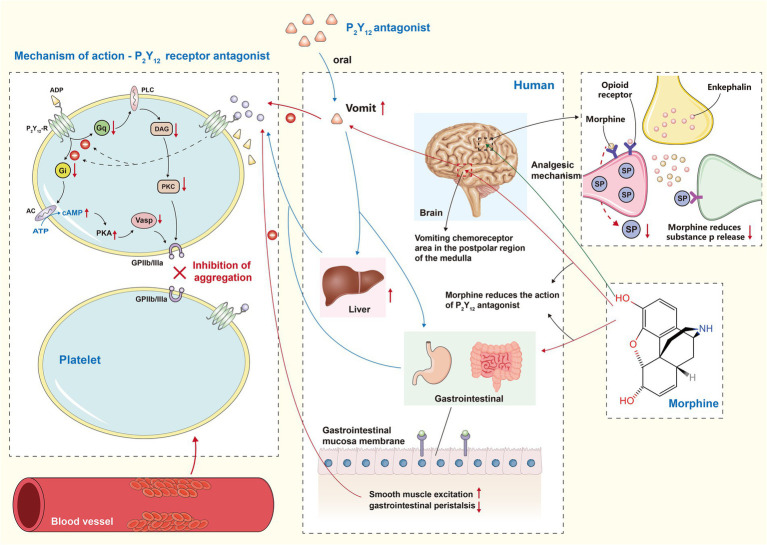
P2Y12 receptor antagonist and morphine interaction effect diagram. Gi, G inhibitory protein; AC, adenylate cyclase; PKA, protein kinase A; Vasp, Vaso dilator stimulatory protein; Gq, Gq protein; PLC, phospho lipase C; DAG, diacylglycerol; PKC, protein kinase C; The left part of the figure represents the mechanism of action of P2Y12 receptor antagonist. They ultimately reduce the stimulation of GPIIb/IIIa by inhibiting both Gi/AC/PKA and Gq/DAG/PKC pathways. The middle part of the figure shows that morphine slows down the absorption of P2Y12 receptor antagonist and reduces the final exposure by delaying gastrointestinal motility and stimulating vomiting. The green arrow in the right part of the figure illustrates the analgesic mechanism of morphine, which simulates endogenous enkephalins enabling a decrease in the release of substance P.

Ischemic chest pain is a common symptom in patients with coronary artery disease. Painful stimuli are thought to increase sympathetic activation, which may lead to an increase in myocardial oxygen consumption. This, in turn, may elevate heart rate and systemic vascular resistance (30). Therefore, restoring the blood supply to ischemic myocardium as soon as possible is the most effective way to relieve pain (31). A large number of patients still do not have immediate access to interventional treatment because of the local medical service. Consequently, having access to effective and rapid pain medication is very important. However, the use of analgesics may lead to a decline in the efficacy of drugs used to treat coronary heart disease. How to use these drugs is worth discussing. Oral P2Y12 receptor inhibitor is a new type of anti-platelet aggregation inhibitor. Combination with aspirin is considered a cornerstone drug for the prevention of thrombotic events in STEMI patients undergoing percutaneous coronary intervention (32). However, among STEMI patients undergoing percutaneous coronary intervention (PCI), due to hemodynamic changes and delayed gastrointestinal absorption, the platelet inhibition induced by oral P2Y12 receptor antagonists will be delayed (33). Morphine or fentanyl will further delay the exposure of P2Y12 inhibitors and reduces their exposure by slowing gastrointestinal motility (Figure 1) (34). In addition, the inhibitory effect of morphine typically lasts for more than 4 h, which may contribute to the poor clinical outcome of patients with STEMI. Therefore, lidocaine can be considered a viable option for analgesia in elderly patients with gastrointestinal diseases. Lidocaine has been found to not diminish the antiplatelet effects of ticagrelor, making it a promising non-opioid pain relief option. Furthermore, the use of lidocaine in patients with STEMI did not uncover an increased likelihood of bradyarrhythmia, tachyarrhythmia or neurotoxicity (35). A clinical study comparing intravenous acetaminophen and fentanyl for pain relief in patients with STEMI found no difference between the two. Plasma Ticagrelor concentration in patients who received intravenous acetaminophen was significantly higher than that in those who received intravenous fentanyl, which further demonstrated the inhibitory effect of fentanyl on P2Y12 antagonists (36). In the meantime, there is no evidence that it is affected by opioid analgesics. Whether or not they can become mainstream pain relief programs remains to be determined.

Platelet reactivity (PR) is considered to be a key pathophysiological factor in the development of ST segment elevation myocardial infarction (STEMI) (37). Previous research has highlighted the capacity of morphine to suppress the activity of P2Y12 receptor antagonists. Then, most studies ignore whether patients have high platelet reactivity (HPR) before treatment, which leads to a lack of understanding of the role that platelet reactivity plays in the outcome and prognosis of patients. Through the research of MarioE Canonico et al., we have been able to gain further insight into the prevalence, clinical characteristics, treatment response and results of STEMI patients with no high platelet reactivity prior to treatment (38). The research demonstrated that 20% of STEMI patients had NHPR, and it took hours for HPR patients to achieve effective platelet inhibition after P2Y12 antagonist load before treatment, leading to a detrimental prognosis in hospital. The wide range of clinical diversity observed in STEMI patients further underlines the importance of providing tailored medication options. For instance, individuals with HPR may benefit from a more advanced and intensive antiplatelet therapy, while avoiding the use of opioid analgesics.

It is uncertain whether drug interactions can have a direct impact on clinical results, and only a few observational studies have been conducted. Etienne Puymirat and his colleagues conducted a one-year assessment of in-hospital complications such as mortality, non-fatal re-myocardial infarction, stroke, stent thrombosis, and bleeding in 2438 patients with STEMI, as part of the 2010 FAST-MI study, in order to assess the impact of prehospital morphine use (39). The findings indicated that there was no statistically significant distinction between those who had pre-hospital morphine and those who had not in terms of 1-year mortality. It is possible that the beneficial effects of morphine on hemodynamics, such as a decrease in heart rate and no major changes in systolic blood pressure, may be counteracting its detrimental effect on the delayed clopidogrel effect, thus resulting in no effect on clinical outcome. Surprisingly, no substantial impact of morphine use on mortality in STEMI patients receiving fibrinolytic therapy was noticed in the research conducted by Cantor et al. (40). Nevertheless, there was a strong association between the ingestion of morphine and the danger of reinfarction at 7 and 30 days (OR = 4.45; *p* = 0.018) and (OR = 1.72; *p* = 0.041). It is not difficult to comprehend why morphine use can impede the antiplatelet effect, as well as why it may be associated with more intense chest pain and more serious conditions in those who require it, such as those with anterior wall infarction and larger infarcts. Remo HM et al. found that the use of morphine was linked to a greater likelihood of short-term cardiac ischemic events (adjusted OR = 1.40; *p* = 0.026), with the primary focus of the study being NSTEACS patients (41).

How to overcome the conflicting effects between analgesic drugs and oral P2Y12 receptor antagonists is of urgent concern. The following strategies are available: Pre-hospital emergency treatment with P2Y12 receptor antagonist (8); A gradually upgraded loading dose regimen for P2Y12 receptor inhibitors (42); The concomitant use of glycoprotein IIb/IIIa inhibitors may be advantageous (43); The simultaneous use of gastrointestinal motility promoting drugs and P2Y12 receptor inhibitors to hasten absorption (44). In addition, cangrelor (an intravenous, reversible platelet P2Y12 receptor antagonist) and selatogrel, a new subcutaneous P2Y12 inhibitor to be used in clinical practice, also need to be considered in the future (45–48). A new reticular meta-analysis found that there was no significant difference between cangrelor, clopidogrel, ticagrelor and prasugrel in reducing the risk of ischemic events (49). Selatogrel is more rapidly absorbed by the body when given intravenously or subcutaneously rather than orally. In the meantime, it is not affected by opioid analgesics.

In conclusion, the effects of morphine, fentanyl, and lidocaine on the antiplatelet effects of P2Y12 receptor antagonists were reviewed. Although pharmacodynamic studies have shown that opioid analgesics can lead to high residual platelet reactivity, however, it is still unclear how this phenomenon affects clinical outcomes. However, it is still advisable to avoid the routine use of opioid analgesics in STEMI patients, unless in specific condition. The advantages of lidocaine include its rapid analgesic effects and lack of inhibition of the antiplatelet effect. However, larger and prospective randomized clinical studies are needed to confirm whether this will become the new mainstream analgesic method.

## Author contributions

HC: conceptualization, writing – original draft, and methodology. HW and LH: writing – review and editing. BL and MK: data curation and validation. LY: supervision, conceptualization, and visualization. All authors contributed to the article and approved the submitted version.

## Funding

This work was supported by the Doctor Start-up fund of Jiangxi provincial People’s Hospital, The First Affiliated Hospital of Nanchang Medical College (No. 19–236).

## Conflict of interest

The authors declare that the research was conducted in the absence of any commercial or financial relationships that could be construed as a potential conflict of interest.

## Publisher’s note

All claims expressed in this article are solely those of the authors and do not necessarily represent those of their affiliated organizations, or those of the publisher, the editors and the reviewers. Any product that may be evaluated in this article, or claim that may be made by its manufacturer, is not guaranteed or endorsed by the publisher.
